# Authentic Leadership and Proactive Behavior: The Role of Psychological Capital and Compassion at Work

**DOI:** 10.3389/fpsyg.2018.02470

**Published:** 2018-12-17

**Authors:** Yixin Hu, Xiao Wu, Zhaobiao Zong, Yilin Xiao, Phil Maguire, Fangzheng Qu, Jing Wei, Dawei Wang

**Affiliations:** ^1^School of Psychology, Shandong Normal University, Jinan, China; ^2^Department of Computer Science, National University of Ireland, Maynooth, China

**Keywords:** authentic leadership, proactive behavior, psychological capital, compassion at work, social information processing

## Abstract

This study, which is based on survey data provided by 445 employees from a Chinese enterprise, examines the impact of authentic leadership on the proactive behavior of subordinates, in particular the mediating effect of subordinate psychological capital and the moderating effect of compassion at work. The results of our structural equation model reveal that: (1) There is a significant positive correlation between authentic leadership and the proactive behavior of subordinates; (2) psychological capital plays a full mediating role between authentic leadership and subordinate proactive behavior; (3) Compassion at work has a moderating effect on the positive relationship between authentic leadership and subordinate psychological capital and proactive behavior.

## Introduction

The modern world is characterized by complex competition, rapid global economic change, and unpredictability (Hong et al., 2016). Given such an uncertain environment, it is not enough for companies to rely solely on employees complying with rules and regulations and following instructions. Instead, organizations need to rely on employees who can engage in proactive behaviors and independently improve the efficiency of their workplace (Belschak et al., 2010; Bindl et al., 2012). Proactive behavior has been shown to support positive outcomes for both individuals and their employers (Bindl and Parker, 2010), such as increased job satisfaction (Anseel et al., 2015), better task performance (Weseler and Niessen, 2016), and superior organizational performance (Saks et al., 2011). As a result, the question of how best to promote proactive behavior is of particular relevance for organizations operating in competitive domains.

Leadership is considered to be an important factor affecting proactive behavior. Fuller et al. (2015) argued that the response of leaders to the proactive behavior of employees influences the enthusiasm of employees to engage in such behavior. Multiple studies have shown that positive leadership, such as transformational leadership, has positive predictive effects on proactive behavior (e.g., Strauss et al., 2009; Belschak and Den Hartog, 2011; Den Hartog and Belschak, 2012; Hong et al., 2016). These studies, however, had a specific focus on transformational leadership and leadership vision incentives (Li and Tian, 2014). As a kind of positive leadership, authentic leadership not only has transformative characteristics, but also the characteristics of honesty, integrity, and loyalty. Because it can facilitate the development of real relationships with subordinates, it can be considered as the “root cause” of other active leadership styles (Avolio and Gardner, 2005). Spitzmuller and Ilies (2010) have proposed that authentic leaders are more likely to be considered as positive transformational leaders by their subordinates. In light of these complex associations, it is worthwhile to investigate the relationship between authentic leadership and proactive behavior to reveal the mechanism behind it (Belschak and Den Hartog, 2010).

Assuming that authentic leadership does indeed have an influence on the proactive behavior of subordinates, the question arises as to how the effect occurs, and what the internal mechanism is. Emerging from positive organizational behavior and positive psychology, the idea of active intrinsic energy plays an important role in explaining the internal mechanism of how leadership style impacts subordinates’ behavior (Sweetman and Luthans, 2010; Shi et al., 2018). In particular, our study examines the effect of subordinates’ psychological capital on the relationship between authentic leadership and proactive employee behavior. Numerous studies have shown that authentic leaders have a positive predictive effect on psychological capital (Ilies et al., 2005; Han and Yang, 2011; Sun, 2013; Zhang, 2014). In addition, the dimensions of efficacy and optimism in psychological capital were found to be positively related to proactive behavior (Axtell et al., 2000; Ashforth et al., 2007; Bledow and Frese, 2009; Fritz and Sonnentag, 2009). Based on this, we speculate that authentic leadership will enhance the psychological capital of subordinates, improve their motivation, and thus promote their proactive behavior.

At the same time, individuals’ perceptions of others and of their organization affect their interpretation of self and leadership behavior, as well as stimulating or inhibiting their psychological energy (Wei and Zhang, 2010). Compassion at work is manifested as a series of positive cognitions, feelings, and behaviors (Kanov et al., 2004). Studies have shown that high compassion at work, as perceived by employees, enables them to gain more intimacy, support, and happiness (Lilius et al., 2008). This in turn makes employees gain more positive psychological energy (e.g., psychological capital). Grant et al. (2008) found that compassion at work can enhance organizational identity and increase organizational citizenship behavior by shaping members’ perceptions of their organization, their colleagues and themselves (Lilius et al., 2008). In light of these findings, we speculate that individuals who experience a high level of compassion at work are more likely to accept authentic leadership behavior. As a consequence, psychological capital energy will be enhanced and proactive behavior will be promoted.

Based on the above, our study carries out the following explorations: firstly, the impact of authentic leadership on proactive behavior is investigated. Secondly, the mediating effect of psychological capital on the relationship between authentic leadership and the proactive behavior of employees is analyzed. Finally, the moderating effect of compassion at work on the process of authentic leadership and proactive employee behavior is examined. It is worth pointing out that most studies view compassion at work as representing a group atmosphere. Nevertheless, organizations cannot simply be characterized as “compassionate” or “non-compassionate” (Kanov et al., 2004; Dutton et al., 2006). Compassion at work, in essence, is how the environment of an organization affects the ability and willingness of an individual to care, not just as an individual, but as a member of a collective (Dutton et al., 2006). Therefore, in this study, compassion at work is regarded as an individual variable.

**FIGURE 1:**
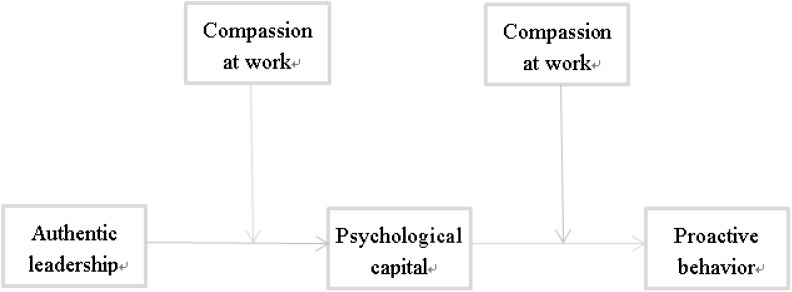
The proposed model of the study.

The research model is shown in Figure 1.

### Proactive Behavior

Grant and Ashford (2008) defined proactive behavior as expected actions that employees use to influence themselves and their environment, including proactive and problem-solving behavior which seeks ways to change a work situation (Frese et al., 1996; Parker et al., 2006; Parker and Collins, 2010). Proactive behavior is somewhat similar to organizational citizenship behavior, though the former is more broadly defined, encompassing both intra-role and extra-role behavior (Bolino and Turnley, 2005). At present, studies on the influencing factors of proactive behavior focus mainly on three aspects: individual factors (e.g., personality, efficacy, and skill), situational factors (e.g., job autonomy, leadership, and support), and their interaction. For example, some studies have found a significant correlation between role breadth self-efficacy (Nguyen et al., 2017; Wu and Parker, 2017; Yin et al., 2017), proactive personality (Li et al., 2017; Wu et al., 2018), and proactive behavior. Moreover, some studies have shown that situational factors, including work characteristics (Ohly and Fritz, 2010; Shin and Kim, 2015), leadership (Hong et al., 2016; Nascimento et al., 2018), and organizational factors (Ellis et al., 2017), are also of great significance to the generation of proactive behavior. Some interactions of individual and situational factors may also have a positive impact on proactive behavior. For example, it has been found that transformational leadership can improve employees’ role breadth self-efficacy, which has a further positive effect on their proactive behavior (Strauss et al., 2009; Belschak et al., 2010; Den Hartog and Belschak, 2012). This study will focus on individual and situational factors, and further explore the mechanism and conditions that affect proactive behavior.

### Authentic Leadership and Proactive Behavior

As an important leadership style, authentic leadership has recently attracted extensive research attention (Miao et al., 2018). Walumbwa et al. (2008) defined authentic leadership as a leadership style that promotes positive psychological competence and high moral standards in order to foster active self-development among followers. Zhou and Yang (2013) proposed a new four-factor model of authentic leadership based on Walumbwa et al.’s (2008) work, including subordinate orientation, internalized morality, leadership traits, and honesty.

Subordinate orientation implies that leaders help subordinates to achieve their goals, provide necessary guidance and support, and maintain sufficient respect and frankness in the relationship. Internalized morality means that leaders should obey social morality, adhere to their own beliefs and be sure that their behavior at work is consistent with their beliefs and values. Leadership traits refer to a leaders’ level of self-awareness, that is, the understanding of self-extension, shortcomings and multifaceted characteristics. Honesty means that the good behavior of leaders should be consistently maintained, and that their actions should always accord with their words. Our study used the definition and measurement tools of authentic leadership proposed by Zhou and Yang (2013).

Many studies have examined the relationship between leadership and employees’ behavior in organizations. Some of these have shown that authentic leadership leads to positive individual and work-related outcomes, such as creativity and knowledge-sharing behavior (Malik et al., 2016), role performance (Leroy et al., 2015), mental health (Laschinger et al., 2015), and work engagement (Hsieh and Wang, 2015). Parker and Collins (2010) proposed that situational variables, especially leadership, are important factors affecting proactive behavior. Furthermore, Belschak et al. (2010) suggested that future work should focus on the impact of various types of leadership on proactive behavior, one of which is authentic leadership. These studies have revealed a positive correlation between transformational leadership and the proactive behavior of employees (Bettencourt, 2004; Belschak et al., 2010; Den Hartog and Belschak, 2012). Given that authentic leadership is a form of positive transformational leadership (Spitzmuller and Ilies, 2010), we regarded authentic as an antecedent that may impact on proactive employee behavior.

Authentic leaders may have a positive impact on the work behavior of their employees because they support employees’ self-determination, thus enhancing their internal motivation for work (Ilies et al., 2005; Parker and Collins, 2010). Authentic leaders with a high degree of self-awareness and transparency can improve the autonomy of employees (Thompson and Vecchio, 2009). They can also promote the generation of intrinsic motivation through the individual redefinition of work tasks (Salanova and Schaufeli, 2008). Furthermore, the situations created by authentic leadership express more support and provide more opportunities for subordinates. If employees feel more support from their leaders, they are more likely to develop proactive behavior (Parker et al., 2006; Griffin et al., 2007).

When studying the influence of authentic leaders on subordinates’ active behavior, Cui et al. (2015) found that authentic leaders were a significant positive predictor for subordinates’ active behavior. Active behavior is a self-regulating behavior whereby an individual sets goals and implements them actively in the workplace (Bindl and Parker, 2010). Given that this is similar to proactive behavior, we put forward the following hypothesis:

*Hypothesis 1*: Authentic leadership is positively related to proactive behavior.

### Mediating Role of Psychological Capital

With the vigorous development of positive psychology, particularly in the field of human resources management, researchers have begun to focus more on the positive and healthy internal energy of employees, known as psychological capital (Luthans et al., 2006).

Luthans et al. (2007) defined psychological capital as a composite construct encapsulating an individual’s positive psychological state of development. It consists of four dimensions, namely self-efficacy, hope, optimism, and resilience. These dimensions pertain to an individual’s effort to succeed at challenging tasks, to persevere toward goals, to make positive attributions about success, and to bounce back from adversity (Luthans et al., 2007).

Existing studies have shown that leadership is an important antecedent variable of psychological capital. For example, transformational leadership and ethical leadership have a positive impact on psychological capital (Gooty et al., 2009; Pires, 2017; Hu et al., 2018). Authentic leadership is similar in that authentic leaders treat subordinates with care and encourage employees to realize their values (Shamir and Eilam-Shamir, 2005; Zhong et al., 2013).

According to the Job Demand-Resource Model (Bakker and Demerouti, 2007), there are two types of factors that affect the results of work: work requirement and work resource. Authentic leadership, as an effective work resource, can reduce the physical, psychological, social, and organizational costs of work requirement, thereby facilitating the achievement of work goals and promoting individual learning and development (Demerouti et al., 2001).

Authentic leaders are self-confident, hopeful, optimistic, flexible, honest, and have a correct understanding of themselves. They can be recognized by employees through their values, knowledge, and behavior. They act as an example to motivate employees, encourage employees to learn from them and generate more positive self-awareness (Luthans and Avolio, 2003; Shamir and Eilam-Shamir, 2005; Xanthopoulou et al., 2009). Such leaders make employees more optimistic and hopeful, which ultimately becomes self-fulfilling (Avolio et al., 2004). Accordingly, we predict that authentic leadership can promote the development of employees’ psychological capital.

Numerous studies have investigated the outcome variables of psychological capital. Some of these have found that employees with a high level of psychological capital can promote their own positive behavior and organizational development (Larson and Luthans, 2006; Zhong, 2007; Luthans et al., 2008; Verleysen et al., 2015). Psychological capital, as a positive personal resource, can promote employees’ confidence in their own behavior, making them more willing to propose suggestions for the organization (Demerouti et al., 2001). Other studies have shown that psychological capital can promote employees’ positive behavior. First, employees with a high level of psychological capital exhibit more organizational commitment, more participation in the organization (Mohammadi et al., 2016), and more organizational citizenship behavior (Jung and Yoon, 2015), which results in more spontaneous high-return initiatives (Larson and Luthans, 2006; Zhong, 2007; Mohammadi et al., 2016). In addition, employees with high psychological capital are more confident in the company, more optimistic about the future and more hopeful (Wang et al., 2014), so they are likely to make more proactive recommendations for the company. Hsiung (2012) showed that authentic leadership is positively correlated with employees’ voice behavior, a form of proactive behavior.

According to the existing empirical research and associated theory, we speculate that psychological capital can predict the proactive behavior of employees. Thus, we put forward the hypotheses:

*Hypothesis 2:* Authentic leadership is positively related to psychological capital.
*Hypothesis 3:* Psychological capital is positively related to proactive behavior.
*Hypothesis 4:* Psychological capital mediates the positive relationship between authentic leadership and proactive behavior.

### The Moderating Effect of Compassion at Work

Compassion at work originates from concern about colleagues’ pain, evolving into a social process in which all members of the organization identify such pain and disseminate their empathy, and respond in their pain (Li et al., 2014). Compassion at work has attracted the attention of many researchers (Dutton et al., 2014). Most studies have regarded it as an individual perceptual factor and measured the extent to which individuals feel cared about (Hansen and Trank, 2016). The perception of high organizational care by employees can lead to a series of positive outcomes, such as high job satisfaction and commitment to the organization (Lilius et al., 2012). In particular, compassion at work may affect the relationship between authentic leadership and employees’ psychological capital. According to the theory of social information processing, employees are adaptive. They interpret clues provided by the working environment to understand and model their leaders, adjusting their work attitudes, and behaviors according to the information perceived (Salancik and Pfeffer, 1978). When employees feel high levels of compassion, they tend to generate positive emotions (Lilius et al., 2008), which can promote individual self-improvement and psychological development (Frazier, 2009; Breines and Chen, 2012). In such cases, employees are more likely to view their leaders and colleagues as sincere and credible. They will be more willing to accept the influence of authentic leadership, and then promote the development of psychological capital (Chu, 2016). However, if employees feel that the members of their organization are disregardful and believe that their behaviors will produce unfair results, they may develop a sense of distrust of authentic leadership, thus weakening the influence of such leadership on employees’ psychological capital (Tyler and Degoey, 1995; Tyler and Blader, 2000; Sui et al., 2012). Therefore, although authentic leadership has a positive effect on psychological capital, the size of the effect depends on the context in which it is applied. Accordingly, the following hypothesis is proposed:

*Hypothesis 5*: Compassion at work moderates the relationship between authentic leadership and psychological capital.

In an organizational context, compassion at work is also of great importance for employees’ proactive behavior, since this active behavior transcends the boundaries of roles. If employees have a high level of psychological capital and experience a high level of compassion at work, they may be more willing to offer advice to the organization. By contrast, if employees do not feel compassion at work, they are more likely to assume that colleagues or leaders ignore their own efforts, thus dampening enthusiasm and weakening the positive role of psychological capital on proactive behavior (Crant, 2000; Fritz and Sonnentag, 2009).

Following the Job-Requirements-Resource Model, Halbesleben et al. (2014) proposed that whether an individual invests in psychological resources depends on the value of the investment. The experience of a high level of care in an organization can make it easier for employees to invest their psychological resources in the organization. Compassion at work can help employees feel their work is valued (Clark, 1987; Frost et al., 2000; Frost, 2003; Dutton et al., 2014), enhance organizational commitment, and involvement (Grant et al., 2008; Lilius et al., 2008), and promote positive behavior. Therefore, when individual perceptions of compassion at work are high, the possibility of proactive behavior is high, which implies that psychological capital can play a role in promoting employees’ proactive behavior. On the contrary, if employees do not feel compassion at work, they will tend to worry that their proactive behavior might be rejected, thus weakening the relationship between psychological capital and proactive behavior. In light of this reasoning, we propose the following hypothesis:

*Hypothesis 6*: Compassion at work moderates the relationship between psychological capital and proactive behavior.

## Materials and Methods

### Sample and Procedure

We collected the data from a large Chinese communications company through printed surveys. With the support of the company’s HR staff, we randomly invited 488 employees who participated voluntarily. We explained the purpose of the study and noted that they could refuse to participate and that their responses would be kept confidential. Written informed consent was obtained from all participants included in the study. All procedures followed were in accordance with the ethical standards of the Academic Board of Shandong Normal University. The study was reviewed and approved by the Ethics Committee of Shandong Normal University. All data, including demographics (e.g., age, gender, education, work tenure, and position level), authentic leadership, psychological capital, compassion at work, and proactive behavior were collected from the employees. After data collection was complete, we eliminated invalid data or missing data.

We distributed 488 questionnaires and received 445 valid responses. Of the 445 employees, 58.9 percent were female and 73.7 percent were married. In terms of age, 13.7 percent were aged 25 or below, 80.9 percent were between 26 and 35 years old, and 5.3 percent were aged 36 or above. In terms of education, 27.7 percent had high-school education or less, 53.5 percent had some higher education without a bachelor’s degree and 18.8 percent had a bachelor’s degree. In terms of position level, 1.8 percent were in management positions, 96.4 percent were in non-managerial roles and 1.8 percent were in other positions. The average length of tenure at the organization of the employees was 4–6 years. All data were analyzed anonymously.

### Measures

#### Authentic Leadership

Authentic leadership was measured using a 17-item scale developed by Zhou and Yang (2013) based on the content proposed by Xie (2007) and Walumbwa et al. (2008). Its dimensions include the following: (1) Honesty (5 items), e.g., “My immediate superiors never issue false information,” (2) leadership qualities (4 items), e.g., “My immediate superiors are far sighted,” (3) subordinate-oriented (4 items), e.g., “My immediate superiors respect me enough,” and (4) internalized moral perspective (4 items), e.g., “My immediate superiors’ actions are consistent with their own beliefs.” Responders were asked to indicate the leadership behavior of their immediate supervisors on a 5-point Likert scale ranging from 1 (strongly disagree) to 5 (strongly agree).

We conducted a confirmatory factor analysis (CFA) to examine the construct validity of this measure. The results showed that the fit indices were within an acceptable range [χ^2^/*df* = 3.273, CFI = 0.97, TLI = 0.96, RMSEA = 0.07; (1) honesty (CR = 0.93, AVE = 0.78), (2) leadership qualities (CR = 0.91, AVE = 0.73), (3) subordinate-oriented (CR = 0.91, AVE = 0.68), and (4) internalized moral perspective (CR = 0.93, AVE = 0.76); 17 items α = 0.97].

#### Psychological Capital

The Psychological Capital Questionnaire (PCQ) used in this study was adapted from Luthans et al. (2007). The 24-item PCQ has six items for all subscales, namely efficacy, hope, optimism, and resiliency, e.g., “If I should find myself in a jam at work, I could think of many ways to get out of it.” Responses are scored on a 6-point Likert scale ranging from 1 (strongly disagree) to 6 (strongly agree).

We conducted a CFA to examine the construct validity of the 24 items, which was taken as a four-fact model. The results showed that the fit indices were within an acceptable range [χ^2^/*df* = 2.707, CFI = 0.94, TLI = 0.93, RMSEA = 0.06; efficacy (CR = 0.85, AVE = 0.48), hope (CR = 0.88, AVE = 0.55), resiliency (CR = 0.89, AVE = 0.57), and optimism (CR = 0.89, AVE = 0.56); 24 items, α = 0.95].

#### Compassion at Work

Compassion at work was measured by a 3-item scale developed by Lilius et al. (2008), e.g., “I could feel compassion at work from my superiors.” Participants responded on a 5-point Likert scale ranging from 1 (never) to 5 (nearly all the time).

We conducted an analysis of reliability and validity of these 3 items. The results showed that the basic indicators were within an acceptable range (CR = 0.88, AVE = 0.71; 3 items, α = 0.87).

#### Proactive Behavior

A six-item scale revised by Li and Tian (2014) based on Fuller et al. (2012) was used to assess the proactive behavior of employees, e.g., “The subordinate put forward new and more effective ways to work for the organization.” Participants responded on a 6-point Likert scale ranging from 1 (never) to 6 (very frequently).

In the present study, the results of a CFA on the 6 items as a one-factor model were as follows: χ^2^/*df* = 2.478, CFI = 0.99, TLI = 0.98, RMSEA = 0.06; CR = 0.87, AVE = 0.55; 6 items, α = 0.87, which means that it had a good fit with the data.

## Results

### Preliminary Analysis

The collected data were tested for common method bias. Harman’s single-factor test was used with all variables for an Exploratory Factor Analysis (EFA). The method assumes that if a single factor is extracted, or if the explanatory power of a factor is particularly large, then there is a serious common method bias (Zhou and Long, 2004). The results of EFA showed that seven factors were extracted, explaining 67.30 percent of the total variance, with the biggest factor accounting for 24.51 percent of the variance. Although this process didn’t completely exclude the possibility of common method bias, the results showed that the data collected in this study did not have serious common method bias. Means, SD, and correlations are shown in Table 1. All variables have acceptable internal consistency alphas of above 0.70. The results show that authentic leadership is positively related to psychological capital (*r* = 0.45, *p <* 0.01), compassion at work (*r* = 0.62, *p <* 0.01), and proactive behavior (*r* = 0.17, *p <* 0.01). There is a significant relationship between psychological capital and proactive behavior (*r* = 0.38, *p <* 0.01).

**Table 1:** Means, SD, alpha reliabilities, and correlations.

Variable	Mean	*SD*	1	2	3	4
(1) Authentic leadership	3.93	0.81	0.97			
(2) Psychological capital	4.69	0.93	0.45^∗∗^	0.95		
(3) Compassion at work	3.30	0.99	0.62^∗∗^	0.44^∗∗^	0.87	
(4) Proactive behavior	4.28	0.97	0.17^∗∗^	0.38^∗∗^	0.27^∗∗^	0.87


**N* = 445. The diagonal is the internal consistency coefficient. ^∗∗^*p* < 0.01, the same below.*

### Tests of the Hypotheses

The coefficients of path analysis were analyzed by Mplus 7.0 to test the mediating effect of psychological capital in the relationship between authentic leadership and proactive behavior. The results are presented in Table 2. Authentic leadership was found to be positively related to psychological capital (*b* = 0.357, *p <* 0.001), and psychological capital predicted proactive behavior (*b* = 0.311, *p <* 0.001) when the control variables were controlled, thus supporting the mediating role of psychological capital. Meanwhile, authentic leadership had a non-significant effect on proactive behavior (*b* = 0.075, *p >* 0.05) when psychological capital was controlled. In summary, these results support the idea that psychological capital fully mediates the effect of authentic leadership on proactive behavior. Thus, hypotheses 2–4 are supported.

**Table 2:** Results of path analysis.

	Psychological capital			Proactive behavior		
		
	*b*	*SE*	95% IC	*B*	*SE*	95% IC
Intercept	-0.099	0.247	[-0.742, 0.617]	2.571***	0.244	[1.927, 3.129]
Gender	0.204	0.127	[0.000, 0.529]	0.113	0.096	[-0.033, 0.416]
Age	0.001	0.060	[-0.303, 0.073]	-0.035	0.061	[-0.194, 0.129]
Educational level	0.002	0.057	[-0.145, 0.151]	0.107	0.055	[-0.033, 0.259]
Job experience	-0.051	0.042	[-0.157, 0.157]	0.068	0.047	[-0.039, 0.200]
Marriage	-0.109	0.042	[-0.171, 0.055]	-0.017	0.073	[-0.173, 0.233]
Authentic leadership (X)	0.357***	0.058	[0.209, 0.506]	0.075	0.061	[-0.217, 0.090]
Psychological capital (M)				0.311***	0.056	[0.173, 0.460]
Compassion at work (W)	0.209***	0.059	[0.056, 0.362]	0.167***	0.058	[0.013, 0.318]
X × W	0.179***	0.039	[0.066, 0.270]	0.020	0.054	[-0.139, 0.137]
M × W				-0.028	0.052	[-0.157, 0.113]


**N* = 445. ^∗∗∗^*p* < 0.001.*

We posited that compassion at work moderates the relationship between authentic leadership and psychological capital. As predicted, after controlling for the direct effects of authentic leadership, compassion at work and control variables, the interaction between authentic leadership and compassion at work on psychological capital was positive and significant (*b* = 0.179, *p <* 0.001), thus supporting hypothesis 5.

The results presented in Table 2 also reveal that the interaction of authentic leadership and compassion at work with proactive behavior was not significant (*b* = 0.020, *p >* 0.05) after controlling for the direct effects of authentic leadership, compassion at work, psychological capital, and control variables. This suggests that compassion at work doesn’t moderate the effect of authentic leadership on proactive behavior.

Lastly, the results showed that the interaction between psychological capital and compassion at work on proactive behavior was not significant (*b* = -0.028, *p >* 0.05) after controlling for the direct effects of authentic leadership, compassion at work, psychological capital, and control variables.

**FIGURE 2:**
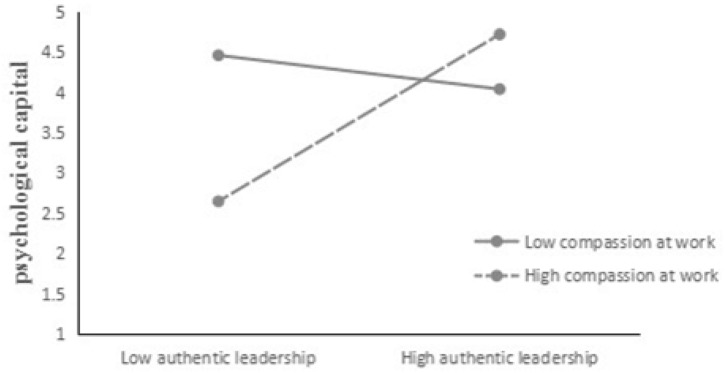
The interaction between authentic leadership and psychological capital on compassion at work.

Figure 2 displays a plot of these results. As expected, the association between authentic leadership and psychological capital is stronger when compassion at work is high. We conducted a simple slope test developed by Aiken and West (1991). The results showed that the conditional indirect effects of authentic leadership on psychological capital were stronger and significant with high compassion at work (1 SD above the mean, *b* = 0.346, *p <* 0.01), but were not significant with low compassion at work (1 SD below the mean, *b* = 0.07, *p >* 0.05).

We examined the extent to which the indirect effects of authentic leadership on psychological capital were contingent upon compassion at work using the PROCESS (Hayes, 2013). As is shown in Table 3, the results revealed that the indirect effect of authentic leadership on proactive behavior through psychological capital was significant when compassion at work was high (+1 SD) but non-significant when compassion at work was low (-1 SD).

**Table 3:** Conditional indirect effects across levels (±1 SD) of compassion at work.

			95% Confidence interval	
			
	Effect	BOOT SE	BOOT LLCI	BOOT ULCI
+1 SD	0.4018^∗^^∗^	0.1413	0.1412	0.6992
Mean	0.7959	0.1568	0.5218	1.1390
-1 SD	1.1899	0.223	0.8013	1.7158


*^∗∗^*p* < 0.01.*

## Discussion

Our study has not only confirmed the positive predictive effects of authentic leadership on proactive behavior but also further revealed a mediating effect of psychological capital in the relationship between authentic leadership and proactive employee behavior. It has also revealed moderating effects of compassion at work in the relationships between authentic leadership and psychological capital, and between psychological capital and proactive behavior. The specific theoretical and practical significance of these findings are discussed in the following sections.

### Theoretical Significance

Firstly, our study has incorporated authentic leadership into the research framework for the first time, confirming that authentic leadership is an important contextual factor for promoting proactive behavior. On the one hand, employees can accept feedback and better understand the direction and prospects of the company through the care, help and motivation of authentic leadership (Kuenzi and Schminke, 2009; Carmeli et al., 2010). On the other hand, in return, employees are more active in contributing to the organization, solving problems proactively, and spontaneously producing proactive behavior.

Secondly, the reason why authentic leadership plays an important role in proactive behavior is that it affects or shapes the relevant psychological state of employees. Based on this, our study further analyzed the mediating effects of psychological capital and examined the relationship between authentic leadership and proactive behavior. As a kind of positive internal psychological energy, we expected that it would have a positive impact on individual behavior.

Our study confirmed that authentic leadership indirectly influences the proactive behavior of employees through influencing psychological capital, thus helping to uncover the “black box” of the process by which authentic leadership influences proactive behavior. Leaders with authentic leadership can evaluate the contribution of subordinates faithfully. They have the vision to show employees the blueprint for the company’s future, thus giving employees hope for the company and for their own work and enhancing their psychological capital energy. All these factors contribute to helping employees invest in their work actively and optimistically, allowing them to maintain their unremitting passion to complete work, and engage in proactive behavior.

Authentic leaders usually follow rules and regulations consciously, treat each employee transparently, do not falsify, treat the upper, and lower equally, and treat all subordinates fairly. As a result, employees can improve their self-efficacy, get timely and effective help from leadership in the face of difficulties, enhance their resilience and psychological capital, and improve their confidence and ability to solve problems constructively. In such a workplace, employees are not only enhanced in their current work tasks, but also improve their work model in the long run, in turn providing good advice and better job performance for the organization (Walumbwa et al., 2011). The results of this study apply not just to in-role behavior, but extend the research to explore extra-role behavior (proactive behavior).

Finally, the effects of leadership depend largely on certain contextual characteristics. With regards to the organization itself, the atmosphere experienced by subordinates often affects the outcome of leadership style. The impact of authentic leadership on employees’ proactive behavior is no exception. In other words, such leadership can achieve nothing without appropriate context. This study combines authentic leadership with relevant theories of compassion at work and examines the contingency effects of employees’ perceived compassion on how authentic leadership impacts the proactive behavior of subordinates. Consistent with existing studies (e.g., Lilius et al., 2008), the results reveal that compassion at work significantly moderates the positive relationship between authentic leaders and the psychological capital of their subordinates. Compared with employees who feel low levels of compassion at work, employees who feel high levels of care are more inclined to identify with authentic leadership behavior and are more willing to dedicate their resources to such positive leaders. Meanwhile, they are more optimistic about their situation and keep high spirits for the future prospects of the organization. In this case, self-efficacy, psychological resilience and psychological capital may be enhanced, leading to more positive proactive behavior.

In sum, our study combined the theory of compassion at work and demonstrated that compassion at work has an important moderating influence on the impact of authentic leadership on the proactive behavior of subordinates.

### Practical Significance

In recent years, dramatic changes in the internal and external work environment have made the issue of employees’ proactive behavior increasingly pertinent (Frese, 2008). Fostering more flexible and proactive work behavior among employees is of unquestionable benefit to an organization for coping with competition, gaining advantage, and succeeding in a dynamic environment (Batistič et al., 2016). Businesses are increasingly dependent on the initiative of its members to discover and solve problems (Frese et al., 1996; Crant, 2000). As employees’ work tasks become more dynamic and more demanding of autonomy, they must perform more actively and must independently create opportunities for self-development.

Our study found that authentic leaders have a significant positive effect on the proactive behavior of subordinates. This suggests that if the leaders who work in various positions of an enterprise want their subordinates to show more proactive behavior, those leaders must themselves fully recognize the positive role of authentic leadership. In daily management, leaders should adjust by eliminating the aspects of their leadership behavior that are detrimental to subordinates’ mental health (e.g., degrading a subordinate’s ability, expressing contempt for a subordinate’s contribution). In addition, leaders should aim to establish individual authentic leadership behavior to better regulate the behavior of their followers, for example, by shaping positive leadership traits, focusing on the needs of their subordinates, constructing impartial institutions, and empowering their subordinates in specific contexts (Farh et al., 2006). Furthermore, in order to promote employees’ proactive behavior, leaders should learn how to provide employees with an opportunity to participate in decision-making processes, to share information with them, and to encourage the sharing of opinions. In light of the importance of authentic leadership, we advise employers to selectively recruit candidates with high levels of authentic leadership as administrators. This can be achieved by formulating relevant policies and using authentic leadership tools. Organizations should formulate a training system for leaders and set training courses to develop talent and cultivate authentic leadership characteristics.

This study also revealed a mediating effect of psychological capital, which indicates that an important prerequisite for subordinates to engage in proactive behavior is to have high levels of psychological capital. Leaders should strengthen their awareness of the importance of psychological capital and seek to enhance it among their staff. Employees themselves should also be selected to hold a high level of psychological capital, which organizations should further develop after they join the company. Through micro-interventions and short-term training courses (Luthans et al., 2008), employees’ psychological capital and proactive behavior can be cultivated, leading to enhancements in organizational performance. Activities can be held to improve employees’ psychological capital (e.g., offering relevant books, inviting experts to give lectures). At the same time, leaders at all levels can enhance the quality of their authentic leadership by expressing compassion and care for their subordinates, thus stimulating proactive behavior.

In this study, we discovered a moderating role of compassion at work, which indicates that compassion at work is an important factor that improves subordinates’ psychological capital. In the past, some Chinese organizations relied on authoritarian systems to restrict subordinates. The realization that subordinates might voluntarily take the initiative and devote themselves to the development of their organization has undermined this approach, ushering in a process of modernization and popularization of Western management theories. Currently in China, many young employees are no longer positive (or even negative) about the value of obeying authority. This suggests that in practice, managers should pay attention to the actual needs of the employees and establish a tolerant organizational culture. They should also implement the Employee Assistance Program (EAP), a free benefit program provided by enterprises to their employees. The EAP aims to solve employees’ difficulties in work and life, create a good organizational atmosphere, and improve the level of compassion experienced at work (Hur et al., 2016). At the same time, leaders must also set an example to other members of the organization, leading to the development of a caring atmosphere. In such organizations, employees feel as if they are truly cared for. This promotes their positive emotions, leads to high levels of psychological capital, and increases proactive behavior.

Finally, our study has important practical relevance to young leaders. Young leaders have little experience in management, making it more important to master such skills quickly. In light of our findings, we suggest that young leaders should spend time and make efforts to build a safe working environment through communication and maintaining their behavioral integrity (Liu et al., 2015; Xu et al., 2017). At the same time, they should pay attention to promoting psychological capital ability through expressing compassion at work. When employees enjoy high levels of psychological capital and receive compassion in their organization, their levels of proactive behavior will be enhanced, leading to favorable individual outcomes such as task performance and greater career success, all of which in turn positively contribute to organizational performance (Batistič et al., 2016).

### Limitations and Future Orientation

Although our research has obtained useful findings, there are some associated limitations that might be addressed by future research.

Firstly, this research relied only on horizontal data. Different designs, such as longitudinal studies, may be used in future studies to better understand the relationship between authentic leadership and employees’ proactive behavior over time.

Secondly, although we strictly controlled the testing process, all the data came from the self-reporting of employees, suggesting a possible common method bias. In the future, proactive behavior should be rated by other reporters such as leaders and colleagues.

Finally, in the data analysis of the research hypotheses, although the demographic variables were considered as control variables, these variables all applied at the individual level. In addition, although we regarded compassion at work as an individual variable, it also undeniably has the characteristics of an organizational variable, which should be clearly distinguished in future studies. Variables relating to organization type, such as company size, and corporate structure, should also be considered.

## Conclusion

Our study aimed to explore the effects of authentic leadership on the proactive behavior of subordinates, in particular the mediating effect of psychological capital and the moderating effect of compassion at work. In summary, this study found that: (1) There is a significant positive correlation between authentic leadership and the proactive behavior of subordinates; (2) Psychological capital plays a full mediating role between authentic leadership and subordinate proactive behavior; and (3) Compassion at work has a significant moderating effect on the positive relationship between authentic leadership and subordinates’ psychological capital and proactive behavior. When the level of compassion at work is high, authentic leadership has a positive effect on psychological capital; when the level of compassion at work is low, authentic leadership has no effect on psychological capital.

## Ethics Statement

All procedures performed in studies involving human participants were in accordance with the ethical standards of the Academic Board of Shandong Normal University and with the 1964 Helsinki Declaration and its later amendments or comparable ethical standards. Informed consent was obtained from all individual participants included in the study.

## Author Contributions

YH and DW designed the study and wrote the paper. XW and ZZ collected the data and wrote the paper. FQ and JW analyzed the data. YX and PM revised and edited the manuscript.

## Conflict of Interest Statement

The authors declare that the research was conducted in the absence of any commercial or financial relationships that could be construed as a potential conflict of interest.
